# Risk of newly developed atrial fibrillation by alcohol consumption differs according to genetic predisposition to alcohol metabolism: a large-scale cohort study with UK Biobank

**DOI:** 10.1186/s12916-023-03229-3

**Published:** 2023-12-21

**Authors:** Chan Soon Park, Jaewon Choi, JungMin Choi, Kyung-Yeon Lee, Hyo-Jeong Ahn, Soonil Kwon, So-Ryoung Lee, Eue-Keun Choi, Soo Heon Kwak, Seil Oh

**Affiliations:** 1 Cardiovascular Center, Seoul National University Hospital, Seoul, Republic of Korea; 2 Division of Data Science Research, Innovative Biomedical Technology Research Institute, Seoul National University Hospital, Seoul, Republic of Korea; 3 Department of Internal Medicine, Seoul National University College of Medicine, Seoul, Republic of Korea

**Keywords:** Atrial fibrillation, Alcohol consumption, Genetic predisposition to disease, Prognosis

## Abstract

**Background:**

The predictive relationship between mild-to-moderate alcohol consumption and the risk of incident atrial fibrillation (AF) remains controversial.

**Objective:**

We investigated whether the relationship between alcohol consumption and the risk of incident AF could be associated with the genetic predisposition to alcohol metabolism.

**Methods:**

A total of 399,329 subjects with genetic data from the UK Biobank database, enrolled between 2006 and 2010, were identified and followed for incident AF until 2021. Genetic predisposition to alcohol metabolism was stratified according to the polygenic risk score (PRS) tertiles. Alcohol consumption was categorized as non-drinkers, mild-to-moderate drinkers (< 30 g/day), and heavy drinkers (≥ 30 g/day).

**Results:**

During the follow-up (median 12.2 years), 19,237 cases of AF occurred. When stratified by PRS tertiles, there was a significant relationship between genetic predisposition to alcohol metabolism and actual alcohol consumption habits (*P* < 0.001). Mild-to-moderate drinkers showed a decreased risk of AF (HR 0.96, 95% CI 0.92–0.99), and heavy drinkers showed an increased risk of AF (HR 1.06, 95% CI 1.02–1.10) compared to non-drinkers. When stratified according to PRS tertiles for genetic predisposition to alcohol metabolism, mild-to-moderate drinkers had equivalent AF risks, and heavy drinkers showed increased AF risk in the low PRS tertile group. However, mild-to-moderate drinkers had decreased AF risks and heavy drinkers showed similar risks of AF in the middle/high PRS tertile groups.

**Conclusions:**

Differential associations between alcohol consumption habits and incident AF across genetic predisposition to alcohol metabolism were observed; individuals with genetic predisposition to low alcohol metabolism were more susceptible to AF.

**Supplementary Information:**

The online version contains supplementary material available at 10.1186/s12916-023-03229-3.

## Background

Atrial fibrillation (AF) is one of the most common clinical arrhythmias and a major cause of stroke, dementia, heart failure, and mortality worldwide [1–3]. For this reason, many attempts have been made to improve the clinical prognosis of AF patients, including refining clinical risk stratification for predicting stroke and thromboembolism, such as CHA_2_DS_2_-VASc scores [4]. In addition, interventional treatment of AF with catheter ablation has become the cornerstone of AF management [5]. Despite these advances, recent studies have shown unacceptably high prevalence and incidence of AF [6, 7], indicating the need for tailored prevention strategies to improve clinical outcomes further.

Various studies have demonstrated that unhealthy lifestyle behaviors such as smoking, obesity, and a lack of regular physical activity are associated with a significantly increased risk of AF [8–12]. Moreover, the risk of cardiovascular diseases such as coronary artery disease and heart failure had a J-shaped association with alcohol consumption [13, 14]. However, reports are contradictory as to whether mild or moderate alcohol consumption are associated with a decreased risk of incident AF [15–18]. This may be due to unmeasured contributing factors that may influence the association between alcohol consumption and the risk of AF. It has been reported that heterogeneity of genetic traits for alcohol metabolism could play a pivotal role in the genetic liability to alcohol consumption habits [19]. Polymorphisms of genes related to alcohol metabolism, such as alcohol dehydrogenase and aldehyde dehydrogenase, could affect differential response after alcohol consumption [20, 21] as well as differential alcohol consumption habits [19, 22]. Therefore, it could be speculated that the genetic predisposition to alcohol metabolism may alter the association between alcohol consumption habits and the risk of incident AF; if an individual has low levels of alcohol metabolic capacity, they might experience more side effects than beneficial effects from alcohol consumption and become less likely to drink alcohol. Unfortunately, there is little data exploring the association between genetic predisposition to alcohol metabolism, alcohol consumption habits, and the risk of AF.

This study aimed to verify whether the association between alcohol consumption habits and incident AF risk differs according to genetic predisposition to alcohol metabolism, using a large prospective cohort from the UK Biobank.

## Methods

### Ethical statement and data availability

This study was conducted in accordance with the principles of the Declaration of Helsinki and approved by the Institutional Review Board (IRB No. E-2203-005-1302). The need for informed consent was waived as anonymized data were used. For reasonable requests, data are available through approval and oversight by the UK Biobank. The UK Biobank has approval from the North West Multi-centre Research Ethics Committee as a Research Tissue Bank approval.

### Data source and study population

The UK Biobank is a large-scale prospective cohort study of approximately 500,000 volunteers enrolled from 2006 to 2010 in the UK. The detailed design and preliminary results have been published elsewhere [23]. In brief, the UK Biobank database includes individual demographic information, diagnostic history, and genomic data. All participants signed a written informed consent form, and the UK Biobank received ethical approval from the National Health Service Research Ethics Service (reference 11/NW/0382).

**Fig. 1:**
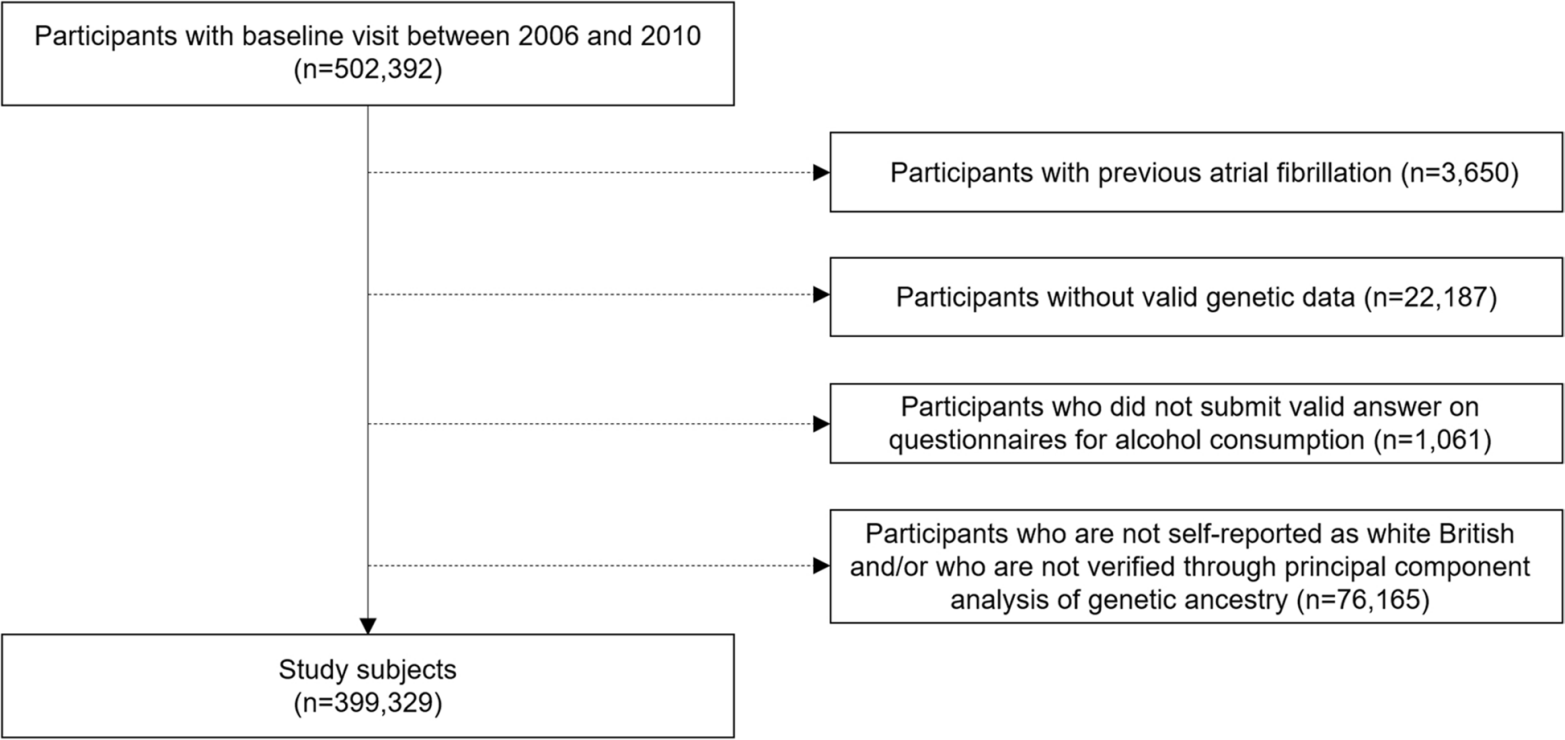
Study population. Study design using data from UK Biobank

The study design is illustrated in Fig. 1. In total, 502,392 individuals enrolled between 2006 and 2010 were identified. Subjects with a history of AF before enrollment (*n* = 3650) were excluded. Moreover, individuals without valid genetic data (*n* = 22,187) and those without valid answers to alcohol consumption questionnaires (*n* = 1061) were also excluded. Among these, we conducted this study among individuals in the white British population who self-reported as white British and were verified through principal component analysis of genetic ancestry. A final total of 399,329 individuals were included in this study and followed up until 2021. The index date was the baseline visit to the UK Biobank.

### Definitions of alcohol consumption habit and genetic predisposition to alcohol metabolism

Using the UK Biobank database, the average alcohol intake per day (g/day) was calculated to evaluate alcohol consumption, and the participants were subsequently categorized into non-drinkers, mild-to-moderate drinkers (<30 g/day), and heavy drinkers (≥30 g/day).

Participants in the UK Biobank were genotyped using the Affymetrix UK Biobank Lung Exome Evaluation (UK BiLEVE) Axiom array or the Affymetrix UK Biobank Axiom array. As previously reported, the UK Biobank team performed central quality control and imputation [24]. To define genetic predisposition to alcohol metabolism, we calculated polygenic risk score (PRS) as the sum of the single nucleotide polymorphism (SNP)’s allele dosages of risk-increasing alleles weighted by their reported log odds ratios. SNPs associated with alcohol metabolism were identified from an existing large genome-wide association study from European ancestry (Supplemental Table 1) [19], which has been explored in other studies [25]. To compare effect sizes corresponding to the continuous PRS, we transformed each PRS of the corresponding analytical data set to the standard normal distribution (Supplemental Fig. 1). According to the PRS, we stratified subjects into three groups based on tertiles: the low PRS tertile group implies a genetic predisposition of slower alcohol metabolism, while the high PRS tertile group implies a genetic predisposition of faster alcohol metabolism.

### Definitions of covariates and the study endpoint

Demographic data, anthropometric data, and medical history of hypertension, diabetes mellitus, myocardial infarction, dyslipidemia, chronic kidney disease, heart failure, and stroke were obtained. In addition, data on smoking history were collected. Smoking history was assessed using standardized questionnaires. Detailed definitions of comorbidities are provided in Supplemental Table 2.

Newly diagnosed AF was defined as the study endpoint. The endpoint was defined based on ICD-10 codes (I48). The follow-up duration was defined as the interval between the index date and the occurrence of incident AF.

### Statistical analysis

Data are presented as numbers and frequencies for categorical variables and mean ± standard deviation or median with interquartile range for continuous variables. For categorical variables, the chi-square test or Fisher’s exact test was used as appropriate. One-way analysis of variance was used to analyze continuous variables between two or more groups. The annual event incidence rate (aIR) was calculated as the number of events per 1000 person-years (PY). Multivariate Cox proportional hazard regression models were used to estimate hazard ratios (HRs) and corresponding 95% confidence intervals (CIs) for the associations between genetic predisposition to alcohol metabolism, real alcohol consumption habits, and the risk of incident AF. Variables that initially achieved *P* < 0.2 in the univariate Cox regression analysis or those with clinical relevance in terms of AF risks were included in a multivariable model (Supplemental Table 3). The multivariable models were adjusted for covariates, including age, sex, previous history of hypertension, diabetes mellitus, myocardial infarction, dyslipidemia, chronic kidney disease, heart failure, stroke, and PRS tertile for alcohol metabolism or alcohol consumption habit, respectively. Subgroup analyses were conducted according to the PRS tertiles for alcohol metabolism and alcohol consumption habits using Cox models. The joint association was demonstrated, with individuals who had heavy drinking habits and low PRS tertile serving as the reference group. We further investigated whether the effects of genetic predisposition to alcohol metabolism could be associated with the risks of incident AF across varying levels of alcohol consumption, using structural equation model (SEM) analysis. With SEM, we aimed to explore the complex association between genetic predisposition, alcohol consumption, and risks of incident AF. A two-sided *P* value of <0.05 was considered statistically significant. Statistical analyses were performed using R programming version 4.2.1 (The R Foundation for Statistical Computing, Vienna, Austria). We manipulated the imputed genotype data and calculated the individual-level polygenic risk score using PLINK version 1.90 (https://www.cog-genomics.org/plink/) [26].

## Results

### Baseline characteristics of the study population

In total, 399,329 individuals without previous history of AF (mean age 56.8 ± 8.0 years; men, 181,981 [45.6%]) were analyzed in this study. Hypertension was diagnosed in 104,879 individuals (26.3%), diabetes mellitus in 16,148 individuals (4.0%), myocardial infarction in 21,369 individuals (5.4%), dyslipidemia in 47,650 individuals (11.9%), chronic kidney disease in 16,695 individuals (4.2%), heart failure in 12,550 individuals (3.1%), and stroke in 8455 individuals (2.1%). More than half of the included participants were never smokers (54.7%), and approximately one-third were ex-smokers (35.0%).

Based on alcohol consumption habits, individuals were classified into three groups: non-drinkers, mild-to-moderate drinkers, and heavy drinkers. The baseline characteristics of each group are presented in Table 1. Heavy drinkers showed male preponderance, had a more frequent history of hypertension and dyslipidemia and substantially higher rates of smoking than non-drinkers and mild-to-moderate drinkers. They also showed a genetic predisposition to higher alcohol metabolism, as calculated by the PRS. In contrast, non-drinkers seemed to have a greater history of diabetes mellitus than other groups. The baseline characteristics classified by a genetic predisposition to alcohol metabolism according to PRS are shown in Table 2. Individuals with a higher PRS for alcohol metabolism tended to consume more alcohol.

**Table 1:** Baseline characteristics according to alcohol consumption habit

	**Non-drinkers (*n* = 114,528)**	**Mild-to-moderate drinkers (*n* = 207,254)**	**Heavy drinkers (*n* = 77,547)**	***P*-value**
Demographic data				
Age, years	57.0±8.2	56.9±8.0	56.4±79	<0.001
Sex, %	36,978 (32.3)	83,621 (40.3)	61,382 (79.2)	<0.001
BMI, kg/m^2^	28.2±5.5	26.7±4.4	27.8±4.2	<0.001
Past medical history, %				
Hypertension	32,398 (28.3)	48,433 (23.4)	24,048 (31.0)	<0.001
Diabetes mellitus	7014 (6.1)	5998 (2.9)	3336 (4.0)	<0.001
Myocardial infarction	6993 (6.1)	9237 (4.5)	5139 (6.6)	<0.001
Dyslipidemia	14,528 (12.7)	21,954 (10.6)	1168 (14.4)	<0.001
Chronic kidney disease	6791 (5.9)	7182 (3.5)	2722 (3.5)	<0.001
Heart failure	4528 (4.0)	5134 (2.5)	2888 (3.7)	<0.001
Stroke	2689 (2.3)	3823 (1.8)	1943 (2.5)	<0.001
Physical examination				
SBP, mmHg	137.0±18.8	137.4±18.5	142.8±18.0	<0.001
DBP, mmHg	81.3±10.1	81.8±9.9	85.3±10.0	<0.001
Smoking, %				<0.001
Never smoker	69,351 (60.8)	120,481 (58.3)	28,467 (37.0)	
Ex-smoker	32,184 (28.2)	71,657 (34.7)	35,468 (46.1)	
Current smoker	12,514 (11.0)	14,508 (7.0)	13,056 (17.0)	
PRS for alcohol use	0.011±0.021	0.012±0.020	0.013±0.019	<0.001

*BMI* Body mass index, *PRS* Polygenic risk score, *S(D)BP* Systolic (diastolic) blood pressure

**Table 2:** Baseline characteristics classified by genetic predisposition to alcohol metabolism according to PRS

	**Low tertile (*n* = 133,054)**	**Middle tertile (*n* = 132,363)**	**High tertile (*n* = 133,912)**	***P*-value**
Demographic data				
Age, years	56.9±8.0	56.8±8.0	56.8±8.0	0.015
Sex, %	60,811(45.7)	60,469 (45.7)	60,701 (45.3)	<0.091
BMI, kg/m^2^	27.3±4.7	27.4±4.7	27.4±4.8	<0.001
Past medical history, %				
Hypertension	34,487 (25.9)	34,950 (26.4)	35,442 (26.5)	0.002
Diabetes mellitus	5273 (4.0)	5340 (4.0)	5535 (4.1)	0.081
Myocardial infarction	7010 (5.3)	6993 (5.3)	7366 (5.5)	0.012
Dyslipidemia	15,740 (11.8)	15,971 (12.1)	15,939 (11.9)	0.157
Chronic kidney disease	5644 (4.2)	5505 (4.2)	5546 (4.1)	0.384
Heart failure	4132 (3.1)	4183 (3.2)	4235 (#.2)	0.634
Stroke	2744 (2.1)	2836 (2.1)	2875 (2.1)	0.233
Physical examination				
SBP, mmHg	138.2±18.5	138.4±18.7	138.4±18.7	<0.001
DBP, mmHg	82.2±10.1	82.3±10.1	82.4±10.1	<0.001
Smoking, %				0.169
Never smoker	72,788 (54.9)	72,298 (54.8)	73,213 (54.8)	
Ex-smoker	46,598 (35.2)	46,241 (35.1)	46,770 (35.0)	
Current smoker	13,182 (9.9)	13,388 (10.1)	13,508 (10.1)	
Alcohol consumption, unit/day	2.1±2.7	2.2±2.8	2.3±2.9	<0.001

*BMI* Body mass index, *PRS* Polygenic risk score, *S(D)BP* Systolic (diastolic) blood pressure

### Predictive implication of a genetic predisposition to alcohol metabolism and real alcohol consumption habit for incident AF

During a median follow-up of 12.2 years (interquartile range, 11.5–12.8), there were 19,237 new cases of AF (4.0%). As shown in Table 3, the aIRs of AF were 5.50, 4.73, and 6.90 per 1000 PY for non-drinkers, mild-to-moderate drinkers, and heavy drinkers, respectively. In the univariate analysis, mild-to-moderate drinkers were associated with a lower risk of AF (HR 0.89, 95% CI 0.86–0.92), but heavy drinkers were associated with an increased risk of AF (HR 1.12, 95% CI 1.08–1.16). After adjusting for covariates, mild-to-moderate drinkers showed a decreased risk of AF (HR 0.96, 95% CI 0.92–0.99), while heavy drinkers consistently showed a higher risk of AF (HR 1.06, 95% CI 1.02–1.10). When analyzed as a continuous variable, one alcohol unit increment per day was significantly related to an increased risk of AF (HR 1.01, 95% CI 1.01–1.02).

**Table 3:** Associations of alcohol consumption habit and PRS for alcohol metabolism with atrial fibrillation

**Variables**	**Total number**	**Atrial fibrillation**	**Incidence rates**	**Unadjusted HR (95% CI)**	***P*-value**	**Adjusted HR**^a^** (95% CI)**	***P*-value**
Alcohol consumption habit							
Non-drinkers	114,528	5640	5.50	1 (reference)	<0.001	1 (reference)	<0.001
Mild-to-moderate drinkers	207,254	8781	4.73	0.89 (0.86–0.92)		0.96 (0.92–0.99)	
Heavy drinkers	77,547	4816	6.90	1.12 (1.08–1.16)		1.06 (1.02–1.10)	
Per 1 alcohol unit/day increment				1.02 (1.02–1.03)	<0.001	1.01 (1.01–1.02)	<0.001
Genetic predisposition to alcohol metabolism							
Low tertile	133,054	6259	5.26	1 (reference)	0.837	1 (reference)	0.388
Middle tertile	132,363	6465	5.45	1.01 (0.98–1.05)		1.01 (0.97–1.04)	
High tertile	133,912	6513	5.42	1.00 (0.97–1.04)		0.99 (0.96–1.03)	
Per 1 SD increment in PRS				1.01 (1.00–1.02)	0.221	1.00 (0.99–1.02)	0.551

Follow-up duration was presented as person-years. Incidence rates were presented as per 1000 person-years

*CI* Confidence interval, *HR* Hazard ratio, *PRS* Polygenic risk score, *SD* Standard deviation

^a^Adjusted for age, sex, previous history of hypertension, diabetes mellitus, myocardial infarction, dyslipidemia, chronic kidney disease, heart failure, stroke, and PRS tertile for alcohol metabolism or alcohol consumption habit, respectively

When individuals were stratified according to PRS tertiles, the aIRs of AF were 5.26, 5.45, and 5.42 per 1000 PY for the low, middle, and high tertiles, respectively (Table 3). In the univariate analysis, the middle tertile group (HR 1.01, 95% CI 0.98–1.05) and high tertile group (HR 1.00, 95% CI 0.97–1.04) had similar risks of incident AF compared to the low tertile group. Similarly, the risk of incident AF was consistently equivalent in the middle tertile group (HR 1.01, 95% CI 0.97–1.04) and high tertile group (HR 0.99, 95% CI 0.96–1.03) compared to the low tertile group in the multivariate analysis.

### Subgroup analyses for AF

**Fig. 2:**
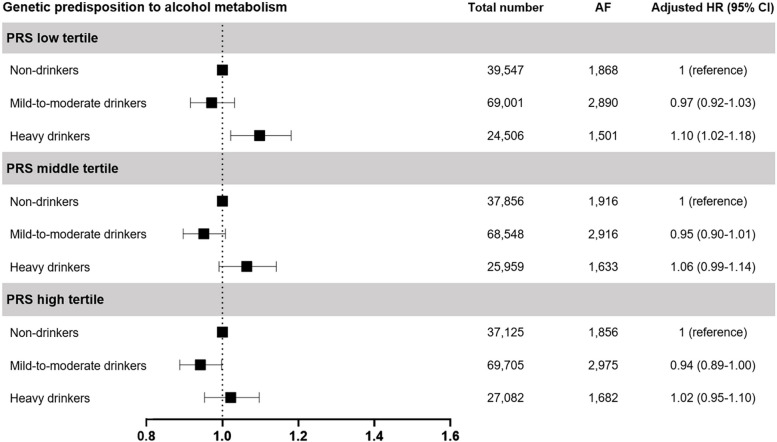
Association between alcohol consumption habit and risks of incident AF according to genetic predisposition to alcohol metabolism. Subgroup analysis according to PRS tertiles for alcohol metabolism was performed to determine whether the prognostic effect of alcohol consumption habits could be modified by genetic predisposition to alcohol metabolism. AF, atrial fibrillation; CI, confidence interval; HR, hazard ratio; PRS, polygenic risk score. *Adjusted for age, sex, previous history of hypertension, diabetes mellitus, myocardial infarction, dyslipidemia, chronic kidney disease, heart failure, and stroke

**Fig. 3:**
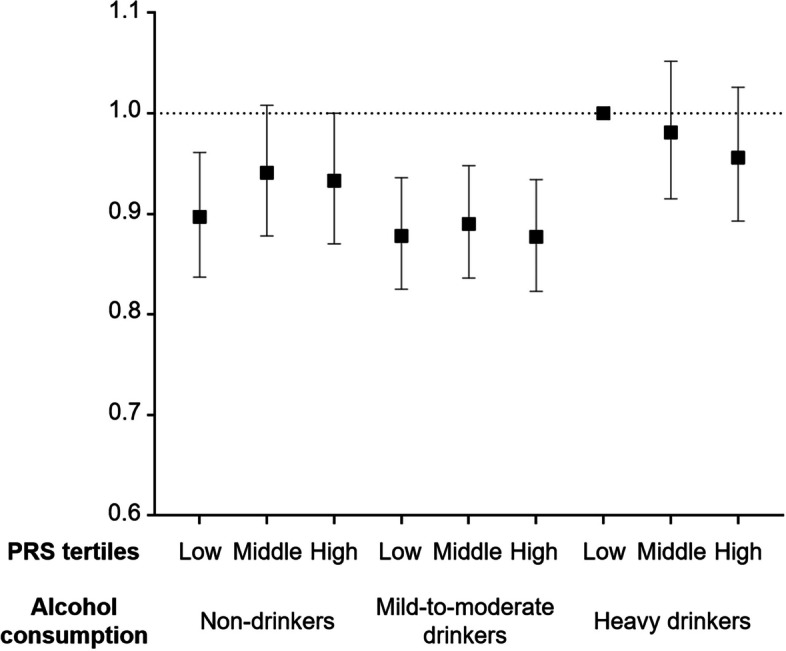
Joint association of alcohol consumption habits and tertiles of genetic predisposition to alcohol metabolism with risks of incident AF. Individuals with heavy drinking habits and low PRS tertile served as the reference group for analysis. Hazard ratios with 95% confidence intervals are displayed as dot and whisker plots, adjusted for covariates. Covariates include age, sex, previous history of hypertension, diabetes mellitus, myocardial infarction, dyslipidemia, chronic kidney disease, heart failure, and stroke

To determine whether the predicted risks of incident AF, based on real alcohol consumption habits, could be modified by a genetic predisposition to alcohol metabolism, we performed a subgroup analysis according to PRS for alcohol metabolism (Fig. 2). In the low PRS tertile group, mild-to-moderate drinkers showed similar risks of AF (HR 0.97, 95% CI 0.92–1.03), whereas heavy drinkers showed a higher AF (HR 1.10, 95% CI 1.02–1.18) than non-drinkers. In contrast, in the middle PRS tertile group, mild-to-moderate drinkers and heavy drinkers showed equivalent risks of AF (HR 0.95, 95% CI 0.90–1.01 and HR 1.06, 95% CI 0.99–1.14), compared to non-drinkers. Similar findings were observed in the high PRS tertile group; mild-to-moderate drinkers and heavy drinkers showed a similar risk of AF (HR 0.94, 95% CI 0.89–1.00 and HR 1.02, 95% CI 0.95–1.10) compared to non-drinkers. The subgroup analysis according to alcohol consumption habits is presented in Supplemental Table 4. When we analyzed joint association across genetic predisposition to alcohol metabolism and alcohol consumption habits, individuals in the low PRS tertile group appeared to be more susceptible to incident AF among drinkers (Fig. 3). In SEM analysis, we found associations between genetic predisposition to alcohol metabolism, alcohol consumption habits, and the risk of AF (Supplemental Table 5) after assessment of the goodness-of-fit parameters indicated an adequate fir for this model (Supplemental Table 6).

## Discussion

**Fig. 4:**
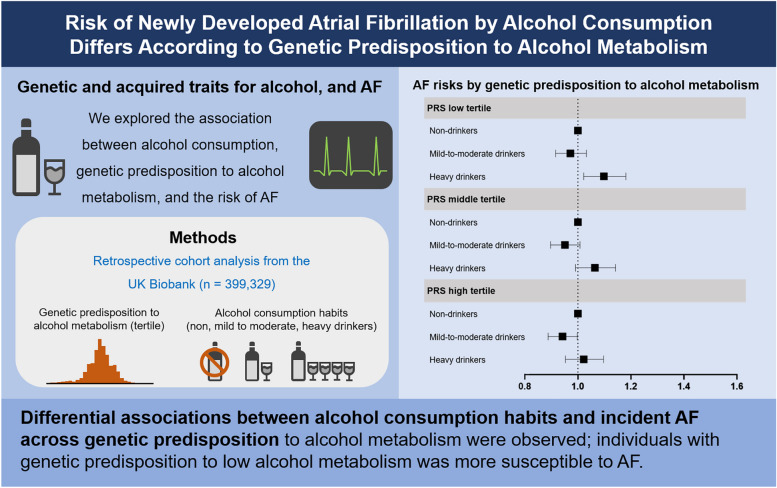
Summarizing illustration. Schematic diagram depicting the association between genetic and acquired traits for alcohol consumption and risks of AF. AF, atrial fibrillation

This study investigated the differential association between actual alcohol consumption habits and incident AF based on genetic predisposition to alcohol metabolism. The major findings are summarized as follows (Fig. 4): First, when the PRS for alcohol metabolism was calculated, there was a significant correlation between genetic predisposition to alcohol metabolism and real alcohol consumption habits. Second, there was significant association between risks of alcohol consumption habits and risks of incident AF. Third, there was no difference in risks of incident AF across PRS tertiles (*P* = 0.221). Fourth, there was a significant interaction between alcohol consumption habits and genetic predisposition to alcohol metabolism for incident AF (*P* for interaction <0.001).

As the number of elderly people increases, the prevalence of AF, the most common clinical arrhythmia, is increasing [1–3]. Since AF is not only a debilitating disease but also could cause comorbidities such as stroke, dementia, and heart failure, there is a demand for effective prevention strategies to mitigate the disease burden. There have been numerous efforts to unveil the association between unhealthy lifestyle behaviors and risks of incident AF, enabling the identification of correctable AF risk factors [8–11]. Alcohol consumption is prevalent worldwide and is an important issue in lifestyle behavior modification [27–29]. A previous study reported that approximately 5% of deaths worldwide could be attributable to alcohol consumption [29]. Due to the detrimental relationship between alcohol consumption and health problems, the association between alcohol consumption and cardiovascular diseases has been studied over the past several decades.

Interestingly, the association between alcohol consumption and cardiovascular diseases is complex [30]. There are reports that mild alcohol consumption might be beneficial to coronary artery disease and heart failure while excessive alcohol consumption could cause grave prognosis [13, 14]. An anti-inflammatory effect and improved insulin sensitivity from small quantities of alcohol consumption were provided as plausible explanations for the aforementioned J-shaped associations between alcohol consumption and cardiovascular diseases [31, 32]. Our study, based on the UK biobank database, also showed nonlinear associations between alcohol consumption and risks of incident AF, as presented in Table 3. Indeed, the association between alcohol consumption and the risk of incident AF remains controversial. Notably, previous studies have reported either a J-shaped association or linear association between alcohol consumption and the risk of AF [12, 15–17]. Several studies utilizing Mendelian Randomization have demonstrated that even mild-to-moderate alcohol consumption could increase the risk of cardiovascular diseases, including AF [33, 34]. However, a nonlinear Mendelian Randomization study has also shown that the increase in risk within the range of mild-to-moderate drinking is minimal [35]. Taken together, the complex association between alcohol consumption and cardiovascular diseases still remains inconclusive and demands further investigation. Meanwhile, we attempted to explore whether the genetic predisposition to alcohol metabolism might influence the association between alcohol consumption and AF risks; we found that there could be individuals who are more susceptible to AF from the same level of alcohol consumption.

Alcohol, especially ethanol, is a substance in alcoholic beverages and is mainly metabolized in the liver [36]. Alcohol metabolism is known to be significantly influenced by genetic factors, including genetic polymorphisms of alcohol dehydrogenase and aldehyde dehydrogenase [36, 37]. Previous studies have reported that genetic polymorphisms in alcohol metabolism are associated with altered levels of acetaldehyde and differential responses to unpleasant experiences after alcohol consumption [20, 21], resulting in increased or decreased susceptibility to habitual alcohol consumption [19, 22]. This suggests genetically decreased alcohol metabolism may result in higher acetaldehyde levels, even after drinking small amounts of alcohol, and could cause flushing syndrome increasing the risk of incident AF [22, 38]. In our data, individuals in the low PRS tertile group showed the lowest alcohol consumption. In contrast, the high PRS tertile group showed the highest alcohol consumption (Table 2). Considering the importance of genetic predisposition to alcohol metabolism and actual alcohol consumption habits, we designed this study to explore the differential predictive relationship of alcohol metabolism according to genetic background. The authors hypothesized that the genetic predisposition to metabolize alcohol could differentiate an individual’s susceptibility to complications and benefits associated with alcohol consumption. If someone has low levels of alcohol metabolic capacity, they might experience relatively more side effects and fewer beneficial effects from alcohol consumption compared to those with high levels of alcohol metabolic capacity. Using the UK Biobank database, we found that the predicted risk of incident AF according to of alcohol consumption differed based on genetic background stratified by PRS tertiles (Fig. 2), which was in line with our hypothesis. Although the genetic ability to metabolize alcohol and/or alcohol metabolites itself was not associated with risks of increased AF as in our report as well as a previous study,^22^ genetic predisposition to alcohol metabolism could alter the relationship between alcohol consumption habits and risks of AF.

To the best of our knowledge, this study is the first to show comprehensive relationships between genetic predisposition to alcohol metabolism, alcohol consumption habits, and risk of incident AF in a sizable cohort with long-term follow-up. This study had two strengths. The UK Biobank is a large population-based prospective cohort study with genotype data. The authors acknowledge that a randomized controlled trial is the best to verify the hypothesis, and an ethical issue could not be avoided in a trial demanding alcohol metabolism. Therefore, a well-designed and well-controlled observational study could be an optimal alternative and provide valuable information. Second, as results of quality control regarding genotype data in the UK Biobank have been reported elsewhere [21], we could explore comprehensive association between genetic predisposition to alcohol metabolism, alcohol consumption habits, and risks of AF with reliable genetic databases.

This study has several limitations. First, alcohol consumption habits might have been altered from baseline during follow-up, for which information was not available. Second, since the study included only individuals in the UK, it is uncertain whether this study could be extrapolated to other ethnicities and countries. However, this large population-based study enabled use of a notably large number of subjects with long-term follow-up, effectively reflecting the phenomenon observed in real-world practice. Additionally, we employed stepwise selections to identify significant confounders, a method that could not exclude the possibility of collider bias during the analysis. Finally, in this study, newly diagnosed AF was defined as the study endpoint based on ICD-10 codes. While continuous ECG monitoring for each individual could potentially detect more cases of AF, it was not feasible in this retrospective cohort study due to its observational nature.

## Conclusions

In this large nationwide study using a prospective registry, we found associations between alcohol consumption habits and incident AF differed according to the genetic predisposition to alcohol metabolism; authors found that PRS might help identify individuals who are more susceptible to developing incident AF than others. Alcohol consumption of more than 30 g/day was associated with increased risks of incident AF in the low PRS tertile group, but the significance was attenuated in the middle/high PRS tertile group. Therefore, alcohol consumption might be more harmful among those with a genetic predisposition to low alcohol metabolism. These findings highlight the importance of assessing genetic predisposition to alcohol metabolism for risk prediction and emphasize the significance of tailored preventive strategies for AF.

### Supplementary Information

**Additional file 1:** **Supplemental Figure 1.** Distribution of Polygenic risk score for alcohol metabolism PRS, polygenic risk score. **Supplemental Table 1.** A list of 10 SNPs known to be associated with alcohol metabolism. **Supplemental Table 2.** Definitions of comorbidities and outcomes. **Supplemental Table 3.** Univariable Cox-proportional hazard regression analysis for overall subjects and for white British subjects. **Supplemental Table 4.** Associations of PRS for alcohol metabolism and atrial fibrillation across alcohol consumption habits. **Supplemental Table 5.** Structural equation models for alcohol consumption, genetic predisposition to alcohol metabolism, and atrial fibrillation. **Supplemental Table 6.** The statistical fit of structural equation models.

## Data Availability

UKB data are available in a public, open access repository. This research has been conducted using the UK Biobank Resource under Application Number 91312. The UK Biobank data are available on application to the UK Biobank (www.ukbiobank.ac.uk/). For reasonable requests, data are available through approval and oversight by the UK Biobank.

## Abbreviations

AF: Atrial fibrillation
aIR: Annual incidence rate
CI: Confidence interval
HR: Hazard ratio
PRS: Polygenic risk score
SNP: Single nucleotide polymorphism
