# Supplementary material for: Risk of newly developed atrial fibrillation by alcohol consumption differs according to genetic predisposition to alcohol metabolism: a large-scale cohort study with UK Biobank
Source: BMC Med. 2023 Dec 21;21:509. doi: 10.1186/s12916-023-03229-3 (PMC10740225; doi:10.1186/s12916-023-03229-3)
Supplement: Supplementary file 1 — Additional file 1: Supplemental Figure 1. Distribution of Polygenic risk score for alcohol metabolism PRS, polygenic risk score. Supplemental Table 1. A list of 10 SNPs known to be associated with alcohol metabolism. Supplemental Table 2. Definitions of comorbidities and outcomes. Supplemental Table 3. Univariable Cox-proportional hazard regression analysis for overall subjects and for white British subjects. Supplemental Table 4. Associations of PRS for alcohol metabolism and atrial fibrillation across alcohol consumption habits. Supplemental Table 5. Structural equation models for alcohol consumption, genetic predisposition to alcohol metabolism, and atrial fibrillation. Supplemental Table 6. The statistical fit of structural equation models. [file 12916_2023_3229_MOESM1_ESM.docx]

**
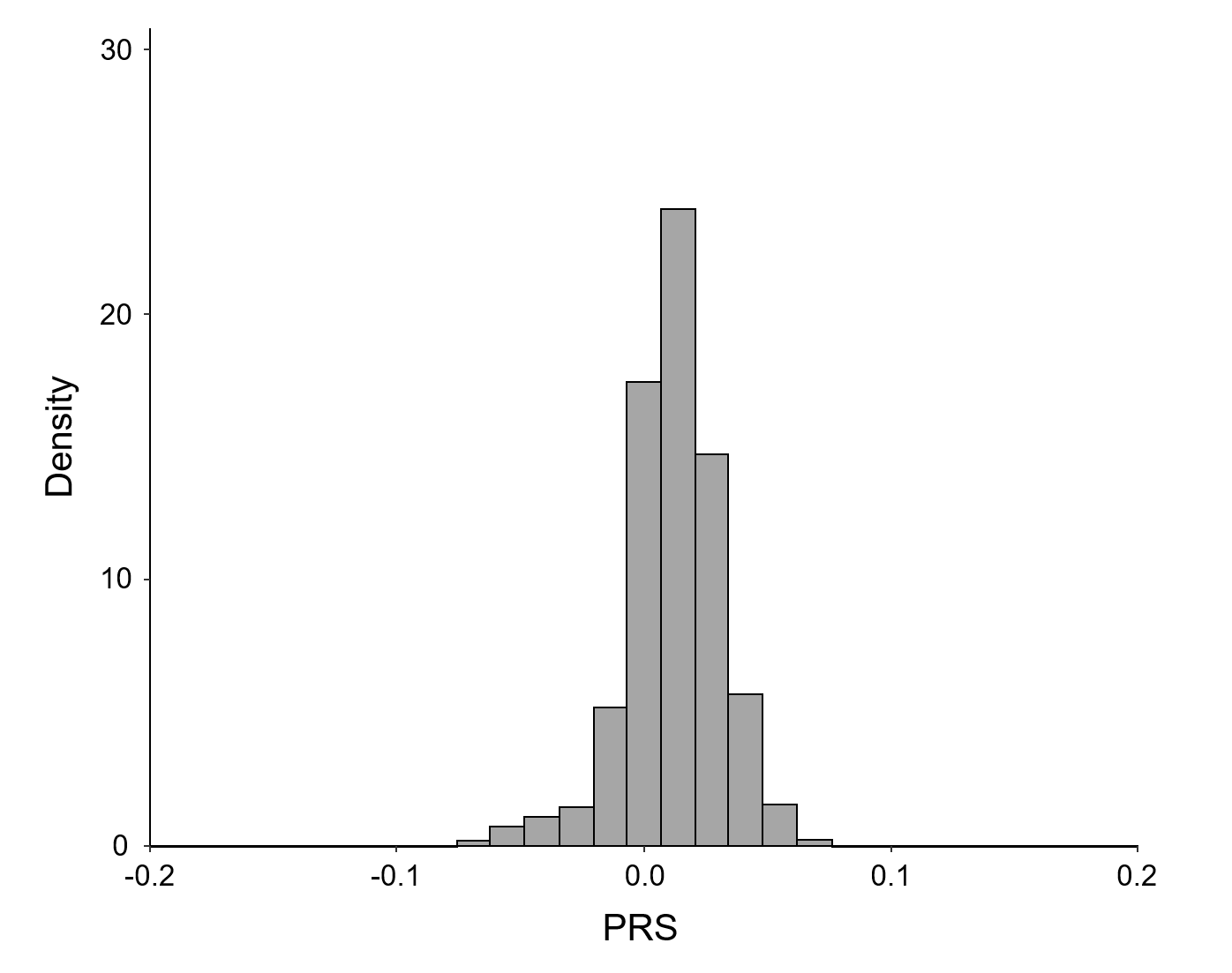
**

**Supplemental Figure 1. Distribution of Polygenic risk score for alcohol metabolism**

PRS, polygenic risk score

**Supplemental Table 1. A list of 10 SNPs known to be associated with alcohol metabolism**

| **SNPs** | **Effect allele** | **Other allele** | **Log (Odds ratio)*** | **P value** | **Effect allele frequency** |
| --- | --- | --- | --- | --- | --- |
| rs1229984 | T | C | -0.72155 | 9.79 × 10^–13^ | 0.040 |
| rs3811802 | G | A | 0.15014 | 2.40 × 10^–8^ | 0.454 |
| rs113659074 | T | G | -0.22314 | 1.54 × 10^–6^ | 0.068 |
| rs1229863 | A | T | 0.13541 | 7.80 × 10^–7^ | 0.174 |
| rs1154445 | G | T | 0.12839 | 1.80 × 10^–7^ | 0.425 |
| rs6827898 | A | G | 0.13541 | 5.21 × 10^–7^ | 0.123 |
| rs894368 | A | C | -0.11991 | 1.93 × 10^–8^ | 0.309 |
| rs79171978 | C | G | 0.18316 | 5.47 × 10^–8^ | 0.099 |
| rs4388946 | C | A | 0.12839 | 7.14 × 10^–7^ | 0.240 |
| rs34929220 | T | C | -0.11317 | 1.02 × 10^–6^ | 0.690 |

*Positive log (Odds ratio) indicate that an allele is associated with an increased risk for the alcohol metabolism, while negative log (Odds ratio) indicate a decreased risk.

SNP, single nucleotide polymorphism

**Supplemental Table 2. Definitions of comorbidities and outcomes**

|  | **ICD-10 codes** |
| --- | --- |
| **Comorbidities** |  |
| Hypertension | I10-I13, I15 |
| Diabetes mellitus | E11-E14 |
| Myocardial infarction | I21, I22 |
| Dyslipidemia | E78 |
| Chronic kidney disease | N18, N19 |
| Heart failure | I50 |
| Stroke | I63, I64 |
| **Outcomes** |  |
| Atrial fibrillation | I48 |

ICD-10, international classification of diseases, Tenth Revision

**Supplemental Table 3. Univariable Cox-proportional hazard regression analysis for overall subjects and for white British subjects**

| **Variables** | **HR** | **95% CI** | **P*-*value** |
| --- | --- | --- | --- |
| Age, years | 1.08 | 1.08-1.09 | <0.001 |
| Male | 1.55 | 1.51-1.60 | <0.001 |
| Hypertension | 1.71 | 1.67-1.76 | <0.001 |
| Diabetes mellitus | 1.74 | 1.66-1.83 | <0.001 |
| Myocardial infarction | 2.08 | 2.00-2.16 | <0.001 |
| Stoke | 2.07 | 1.97-2.17 | <0.001 |
| Dyslipidemia | 1.55 | 1.50-1.61 | <0.001 |
| Chronic kidney disease | 2.37 | 2.28-2.46 | <0.001 |
| Heart failure | 3.20 | 3.09-3.32 | <0.001 |

CI; confidence interval, HR, hazard ratio

**Supplemental table 4. Associations of PRS for alcohol metabolism and atrial fibrillation across alcohol consumption habits**

| **Variables** | **Total number** | **Atrial fibrillation** | **Adjusted HR* (95% CI)** | **P-value** |
| --- | --- | --- | --- | --- |
| Non-drinkers | | | | |
| Low tertile | 39,547 | 1,868 | 1 (reference) | <0.001 |
| Middle tertile | 37,856 | 1,916 | 1.05 (0.99-1.12) |  |
| High tertile | 37,125 | 1,856 | 1.04 (0.98-1.11) |  |
| Mild-to-moderate drinkers | | | | |
| Low tertile | 69,001 | 2,890 | 1 (reference) | <0.001 |
| Middle tertile | 68,548 | 2,916 | 1.02 (0.96-1.07) |  |
| High tertile | 69,705 | 2,975 | 0.99 (0.94-1.05) |  |
| Heavy drinkers | | | | |
| Low tertile | 24,506 | 1,501 | 1 (reference) | <0.001 |
| Middle tertile | 25,959 | 1,633 | 0.98 (0.92-1.05) |  |
| High tertile | 27,082 | 1,682 | 0.96 (0.90-1.03) |  |

CI, confidence interval; HR, hazard ratio; PRS, polygenic risk score

*Adjusted for age, sex, previous history of hypertension, diabetes mellitus, myocardial infarction, dyslipidemia, chronic kidney disease, heart failure, stroke

**Supplemental Table 5. Structural equation models for alcohol consumption, genetic predisposition to alcohol metabolism, and atrial fibrillation**

| **Regressions** | **Standardized coefficient** | **Standardized error** | **Z-value** | **P-value** |
| --- | --- | --- | --- | --- |
| Genetic predisposition to alcohol metabolism ~ alcohol consumption | 0.027 | 0.002 | 14.318 | <0.001 |
| Atrial fibrillation ~ Alcohol consumption | 0.004 | 0.000 | 11.498 | <0.001 |
| Atrial fibrillation ~ genetic predisposition to alcohol metabolism | 0.001 | 0.000 | 2.046 | 0.041 |

CVD, cardiovascular disease

**Supplemental Table 6. The statistical fit of structural equation models**

|  | **SEM** | **Standard criteria** |
| --- | --- | --- |
| CFI | 1.00 | > 0.90 |
| RMSEA | 0.00 | < 0.08 |
| SRMSR | 0.00 | < 0.06 |

CFI, comparative fit index; RMSEA, root mean square error of approximation; SEM, structural equation modeling; SRMSR, standardized root mean square residual
